# Targeting Mitochondrial Reactive Oxygen Species: JP4-039’s Potential as a Cardiovascular Therapeutic

**DOI:** 10.3390/jcm14186465

**Published:** 2025-09-13

**Authors:** Keertana Yalamanchili, Mark Broadwin, Dwight D. Harris, Rayane B. Teixeira, Frank W. Sellke, Peter Wipf, M. Ruhul Abid

**Affiliations:** 1 Cardiovascular Research Center, Rhode Island Hospital, Providence, RI 02903, USA; 2 Division of Cardiothoracic Surgery, Warren Alpert Medical School of Brown University, Providence, RI 02903, USA; 3 Department of Chemistry, University of Pittsburgh, Pittsburgh, PA 15260, USA; 4 Department of Bioengineering, University of Pittsburgh, Pittsburgh, PA 15260, USA

**Keywords:** JP4-039, reactive oxygen species, mitochondrial antioxidants, cardiovascular disease

## Abstract

JP4-039, a mitochondrial-targeted nitroxide, has emerged as a promising candidate in addressing the intricate interplay of reactive oxygen species (ROS) in cardiovascular disease (CVD). Given the substantial mortality and economic burden associated with CVD globally, novel therapeutic strategies targeting oxidative stress hold significant promise. The pathophysiology of CVD encompasses multifaceted mechanisms, including endothelial dysfunction, inflammation, and oxidative stress, where dysregulated ROS levels play a pivotal role. JP4-039, by selectively targeting mitochondrial ROS, offers a targeted approach to mitigate oxidative stress-induced damage in cardiovascular tissue. Current research elucidates the molecular mechanisms underlying JP4-039’s antioxidant properties, including its ability to scavenge superoxide radical anions and mitigate oxidative chain reactions within mitochondria. Moreover, preclinical studies highlight JP4-039’s efficacy in ameliorating CVD-related pathologies, including atherosclerosis and cardiac hypertrophy, through its antioxidative and anti-inflammatory effects. Future milestones in JP4-039 research involve optimizing its pharmacokinetic (PK) properties and exploring potential synergistic effects with existing cardiovascular therapies, followed by advancing into clinical trials.

## 1. Introduction

Cardiovascular disease (CVD) has consistently been the leading cause of death worldwide, resulting in about one in every four deaths in the US [1] and one in every three deaths globally every year [2]. In addition, the US healthcare system spends USD > 400 billion annually on treating CVD and its associated symptoms [3]. Both the high mortality rate of CVD and the economic burden that CVD (and its associated treatments) poses on healthcare systems around the world make it a significant global health concern.

Although the pathophysiology of cardiovascular disease is vast, it can be narrowed down to three main causes: atherosclerosis, coronary artery disease, and high blood pressure in the arteries (arterial hypertension) [4,5]. A common contributor to all three causes of cardiovascular disease is the dysregulation of reactive oxygen species (ROS) levels [6]. With regular cardiovascular function, the equilibrium between ROS levels and biological countermeasures is delicately maintained, avoiding excessive oxidative stress. As a result, a disproportionate increase in ROS or a decrease in antioxidants can disturb the natural redox homeostasis and affect multiple metabolic pathways involved in the cardiovascular system [7].

Mitochondria are both major producers and central regulators of oxidative stress in the heart. Excess mitochondrial ROS (mtROS) drive endothelial dysfunction, and impaired nitric oxide signaling, lipid peroxidation, and the activation of apoptotic cascades, thereby amplifying ischemia–reperfusion injury and adverse ventricular remodeling in coronary artery disease, myocardial infarction, and heart failure [8]. Conventional antioxidants and ROS scavengers have been investigated for cardiovascular therapy, but some have failed in large clinical trials, in part due to their lack of mitochondrial specificity and low bioavailability [9]. This has motivated the development of mitochondria-targeted small molecules designed to neutralize ROS at their primary site of production and restore redox balance more effectively.

Among these agents, JP4-039 is a mitochondria-targeted nitroxide engineered with a gramicidin S-derived peptide isostere that promotes membrane localization and persistence [10]. This design enables JP4-039 to scavenge mtROS and stabilize electron transport chain function. In preclinical studies, JP4-039 has shown protective effects in models of oxidative stress and tissue injury, including reducing apoptosis in irradiated tissues and preserving mitochondrial function in neurons and intestinal cells [10]. These findings highlight JP4-039’s potential relevance to cardiovascular disease, where oxidative stress and impaired mitochondrial function are central drivers of pathology, including endothelial dysfunction and remodeling.

Despite these promising results, important gaps remain. Questions regarding its efficacy across different CVD models, its role in chronic comorbidities such as hypertension and metabolic syndrome, and whether benefits extend beyond structural protection to functional recovery remain unanswered. Moreover, translational challenges persist since pharmacokinetics, tissue bioavailability, and optimal dosing are still not well understood, no human safety or efficacy studies exist, and variable formulations and routes of administration across preclinical studies complicate interpretation. These limitations underscore both the opportunities and barriers to advancing JP4-039 from preclinical testing toward clinical translation.

The primary focus of this review is therefore to summarize the current state of knowledge on JP4-039, discuss its mechanism of action and potential applications in cardiovascular disease, and explore future research directions needed for translation.

## 2. Oxidative Stress and Cardiovascular Disease

### 2.1. ROS Production: Types and Sources of ROS

Reactive oxygen species are a group of highly reactive molecules derived from molecular oxygen [11]. These species play a crucial role in various physiological processes, serving as essential signaling molecules in cellular pathways, regulating gene expression, initiating immune responses, and contributing to redox homeostasis. ROS are also implicated in enzyme-mediated biosynthetic transformations [12]. They can be broadly classified into two main types: free radicals and non-radical species [13]. Free radicals, such as superoxide radical anion (·O_2_^−^) and hydroxyl radical (·OH), contain unpaired valence electrons, making them highly reactive. Non-radical ROS, like hydrogen peroxide (H_2_O_2_) and singlet oxygen (^1^O_2_), lack unpaired electrons but are still potent oxidizing agents. Superoxide radical anion reacts with nitric oxide (·NO) to form peroxynitrite (ONO_2_^−^), a reactive nitrogen species (RNS) that, at low concentration, can also act as a mediator in signaling processes, but, at higher concentration, contributes to tissue damage and oxidative stress [14].

**Figure 1:**
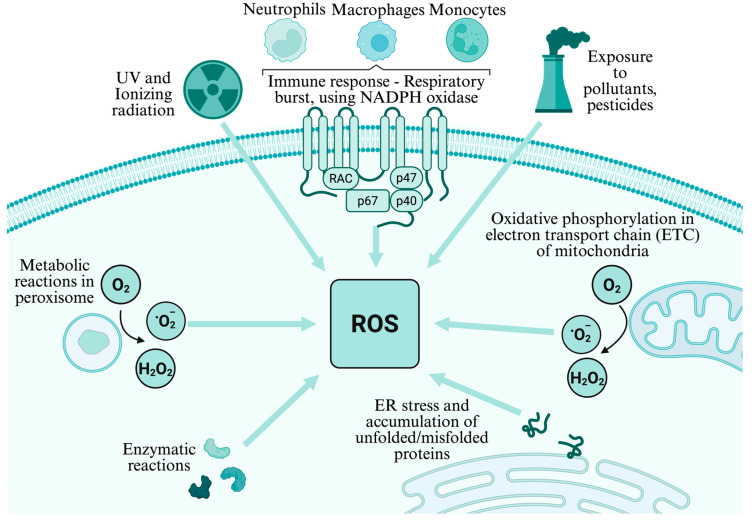
Sources of reactive oxygen species (ROS) in the cell, including the mitochondrial electron transport chain (ETC), peroxisomes, endoplasmic reticulum, and immune responses. These endogenous pathways, along with exogenous sources such as UV and ionizing radiation, and exposure to pollutants and pesticides, contribute to the redox imbalance implicated in cardiovascular disease. Created with BioRender.com. ROS, reactive oxygen species; ETC, electron transport chain; ER, endoplasmic reticulum; O_2_, molecular oxygen; ·O_2_^−^, superoxide anion; H_2_O_2_, hydrogen peroxide; NADPH, nicotinamide adenine dinucleotide phosphate (reduced form); RAC, Ras-related C3 Botulinum Toxin Substrate; p47(phox), Neutrophil Cytosolic Factor 1; p67(phox), Neutrophil Cytosolic Factor 2; and p40(phox), Neutrophil Cytosolic Factor 4.

ROS production is tightly regulated within cells, with enzymes and cellular structures contributing to their generation (Figure 1) [15]. A major endogenous source of ROS includes mitochondrial respiration [16]. Additionally, peroxisomes and the endoplasmic reticulum also contribute to ROS production as part of their normal metabolic functions [17,18].

The mitochondrial electron transport chain (ETC) is the main contributor to endogenous ROS production [16]. During oxidative phosphorylation, electrons leak from the respiratory chain, leading to the formation of superoxide radical anions. This phenomenon is particularly prominent at complex I and complex III of the ETC [19]. Superoxide generated in the mitochondria can then undergo dismutation to form molecular oxygen (O_2_) and hydrogen peroxide, a less reactive, longer-lived ROS [20]. Mitochondrial ROS production is tightly regulated by ETC integrity, antioxidant capacity, enzymatic activity, signaling pathways, and environmental factors. Under normal physiological conditions, these ROS serve as signaling molecules in various cellular processes, including apoptosis and cell proliferation. ETC dysfunction can lead to excessive ROS production, contributing to oxidative stress and potential cellular damage.

Apart from mitochondria, peroxisomes are another cellular organelle involved in ROS production [17]. Peroxisomes are responsible for various metabolic processes, including fatty acid oxidation. During these processes, reactive intermediates are generated, leading to the production of hydrogen peroxide. Unlike mitochondria, peroxisomes lack the machinery to detoxify hydrogen peroxide, making it essential for these organelles to tightly limit ROS production [21]. Furthermore, the endoplasmic reticulum (ER), a multifunctional organelle involved in protein synthesis and folding, is also implicated in ROS generation [18]. ER stress, often triggered by disturbances in cellular homeostasis, can lead to an increase in ROS levels, affecting the redox balance within the cell.

In addition to endogenous cellular processes, the immune response and inflammatory processes represent significant contributors to ROS generation. Neutrophils, macrophages, and monocytes produce ROS as part of their defense mechanisms against pathogens [22]. This phenomenon, known as the respiratory burst or oxidative burst, involves the rapid release of ROS to eliminate invading microorganisms [23]. Neutrophils, for instance, utilize the enzyme nicotinamide adenine dinucleotide phosphate (NADPH) oxidase to generate superoxide radicals during phagocytosis, aiding in the destruction of engulfed pathogens. Similarly, macrophages produce ROS to regulate immune responses and facilitate the clearance of pathogens and cellular debris [24]. The controlled production of ROS by immune cells highlights their physiological role in host defense and immune regulation. Conversely, deficiencies in ROS production, as seen in certain immune disorders like chronic granulomatous disease (CGD), can compromise immune competence, rendering individuals more susceptible to infections [25]. Thus, while ROS are often primarily associated with oxidative stress and disease pathogenesis, their generation by the immune system underscores their essential role in maintaining physiologic functions, particularly in host defense and immunity.

Exogenous factors such as ultraviolet (UV) radiation, ionizing radiation, pollutants, and even physical stressors like intense exercise can also induce ROS production [26,27,28,29,30]. These external stimuli damage cellular components including DNA, proteins, and lipids, thereby amplifying oxidative stress. While less studied than endogenous sources, they interact with mitochondrial and enzymatic ROS production to disrupt redox homeostasis. Understanding the interplay between endogenous and exogenous sources is crucial for unraveling the intricate dynamics of ROS production and their impact on cellular function.

### 2.2. Role of Oxidative Stress in Cardiovascular Disease

Oxidative stress is a pathophysiologic state caused by an imbalance between ROS production and clearance [31]. Several biological mechanisms, such as protein phosphorylation and apoptosis, rely on low intracellular levels of ROS [32,33]. However, when the production of ROS exceeds the ability of the cells to mitigate the effects of ROS through antioxidants or scavenging processes, these essential biological mechanisms are disrupted [34,35]. In atherosclerosis, oxidative stress drives low-density lipoprotein (LDL) oxidation and activates inflammatory pathways such as nuclear factor kappa B (NF-κB) and mitogen-activated protein kinase (MAPK), which in turn promote endothelial dysfunction and plaque progression [36,37,38]. Mitochondrial dysfunction is another key consequence of oxidative stress, as elevated ROS compromise mitochondrial DNA (mtDNA) integrity, reduce adenosine triphosphate (ATP) production, and disrupt antioxidant enzymes like superoxide dismutase 2 (SOD2), catalase, and glutathione peroxidase [39,40]. These processes contribute to the maladaptive remodeling of the heart by influencing hypertrophy, fibrosis, and apoptosis through signaling pathways including protein kinase B (PKB/Akt), MAPK, and TGF-β [41,42,43]. Together, these mechanisms underscore how chronic oxidative stress integrates multiple molecular and structural changes that culminate in cardiovascular disease.

### 2.3. ROS Scavenging: Antioxidants and Scavengers

Antioxidants and ROS scavengers collectively form a dynamic defense network crucial for cellular health by effectively minimizing oxidative stress when ROS are present beyond normal concentrations [44]. The primary difference between antioxidants and ROS scavengers is that antioxidants are generally unspecific reducing agents and quenchers of oxidative molecules or processes, whereas ROS scavengers specifically target and neutralize ROS [45]. Not all antioxidants have the ability to clear ROS efficiently. For example, the antioxidant N-acetylcysteine (NAC) is unable to remove H_2_O_2_ [46].

Antioxidants, encompassing enzymatic and non-enzymatic varieties, neutralize free radicals and prevent oxidative chain reactions by donating electrons or neutralizing reactive intermediates [47]. Superoxide dismutase, catalase, and glutathione peroxidase are notable enzymatic antioxidants that operate within distinct cellular compartments, catalyzing reactions to dismantle harmful ROS [48]. Small-molecule antioxidants, such as vitamins C and E, and ubiquinone (coenzyme Q10), contribute to the defense mechanism by directly interacting with free radicals [49,50]. Metallothioneins and melatonin, which are ROS scavengers, exert their protective effects by sequestering metal ions or neutralizing ROS, respectively [51,52].

#### Global vs. Organelle-Specific Antioxidants

While antioxidants function throughout the cell to reduce oxidative damage, their effectiveness in mitigating disease-related ROS depends significantly on their localization and specificity. Global antioxidants, such as vitamins C and E, NAC, and glutathione, act broadly across the cytosol, plasma membrane, and extracellular space, where they neutralize a wide range of free radicals and reactive intermediates [53,54]. These antioxidants serve essential systemic functions by supporting redox homeostasis and protecting biomolecules like lipids, proteins, and DNA from oxidative modifications. However, they often show limited efficacy in targeting subcellular ROS production sites, particularly the mitochondria, where the ETC is a predominant source of oxidative stress under pathological conditions [55]. To address this limitation, organelle-specific antioxidants have been developed to concentrate in specific intracellular locations, most notably the mitochondria (Table 1).

Mitochondria-targeted antioxidants such as JP4-039, MitoQ, SkQ1, XJB-5-131, SS-31, and MitoTEMPO are designed to traverse lipid membranes and accumulate within mitochondria via different mechanisms, including conjugation with triphenylphosphonium (TPP^+^) cations or gramicidin S-based peptide mimetics [59,62,77,78,79]. These compounds specifically scavenge mitochondrial superoxide and hydrogen peroxide, reducing oxidative damage to mitochondrial DNA, proteins, and membranes, while improving ETC function and ATP production.

Given the varying degrees of mitochondrial localization observed among these agents (Table 1), it is important to emphasize that higher enrichment does not necessarily translate into superior therapeutic potential. For instance, although MitoQ accumulates up to 1000-fold within mitochondria, it has been shown to cause mitochondrial swelling and depolarization in kidney tissue, raising concerns about its safety in long-term use [80]. SkQ1, although highly potent, has been reported to display a narrow therapeutic window, with higher doses causing mitochondrial damage and cell death [81]. SS-31, despite its remarkable ~5000-fold mitochondrial targeting and demonstrated efficacy in preclinical cardiovascular and renal models, has been associated with frequent but mild injection site reactions in clinical studies (erythema, pruritus, and pain), and still lacks large-scale clinical validation for long-term safety and efficacy [82]. In contrast, JP4-039 is a small-molecule antioxidant with moderate mitochondrial targeting, which allows it to scavenge ROS not only within mitochondria but also in other cellular compartments [9]. Importantly, preclinical studies consistently demonstrate a favorable safety profile, with efficacy shown in irradiated and Fanconi anemia mouse models, where JP4-039 reduced mucositis and marrow suppression without evidence of systemic toxicity [83,84]. Taken together, these features position JP4-039 as a complementary and potentially more practical therapeutic option among mitochondrial-targeted antioxidants.

## 3. JP4-039: A Nitroxide-Based ROS Scavenger

### 3.1. Structure and Functional Groups

**Figure 2:**
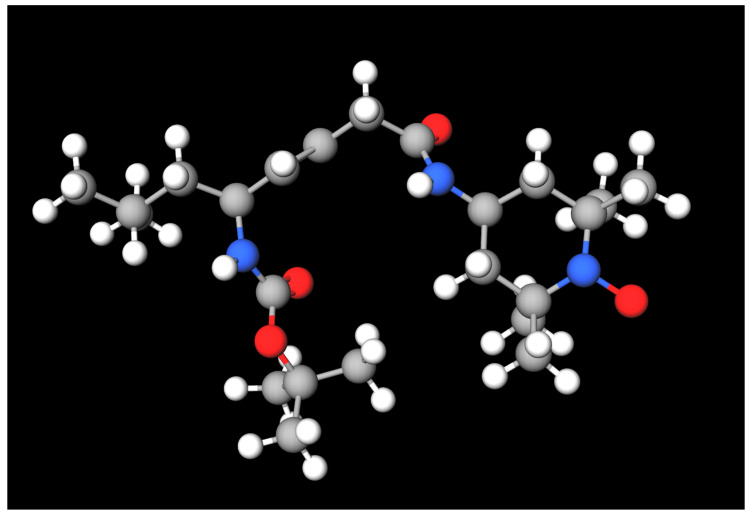
Molecular structure of JP4-039 (PubChem Compound ID: 70894087), where gray atoms represent carbon, white atoms represent hydrogen, red atoms represent oxygen, and blue atoms represent nitrogen. This 3D molecular model was generated and visualized using MolView v2.4 software.

JP4-039, a nitroxide structurally related to XJB-5-131 and 4-amino Tempo (4-AT), is a small-molecule ROS scavenger that maintains mitochondrial redox homeostasis [85]. It was modeled after the structure of gramicidin S, a membrane-associated antibiotic that acts against Gram-positive and Gram-negative bacteria, along with certain types of fungi. Its structure contains a nitroxide free radical moiety as a key functional group responsible for JP4-039’s radical scavenging activity, allowing it to rapidly neutralize ROS (Figure 2) [86].

Compared to a larger and more highly mitochondrial-targeted synthetic nitroxide, XJB-5-131, JP4-039 has a more drug-like character as well as a higher aqueous solubility, while maintaining the key features of XJB-5-131 [87]. JP4-039 has a smaller concentration difference across the mitochondrial membrane than XJB-5-131 and thus remains more available for scavenging ROS formed in other cellular compartments [87]. Specifically, JP4-039 is 30 times more concentrated in the mitochondria versus the cytosol, whereas XJB-5-131 is 600 times more concentrated in the mitochondria versus the cytosol.

### 3.2. Antioxidant Defenses in the Striatum

In a study investigating the effect of JP4-039 on sulfite-induced alterations in antioxidant defenses in the rat brain’s striatum, it was observed that JP4-039, administered through an intraperitoneal injection at 3 μg/g 24 h and 5.0 μg/g 2 h prior to sulfite injection, attenuated reductions in glutathione peroxidase (GPx) and glucose-6-phosphate dehydrogenase (G6PDH) [9]. The concentrations of GPx and G6PDH were measured spectroscopically by performing an assay monitoring NADPH oxidation in the presence of striatum supernatant. In addition, treatment with JP4-039 prevented a sulfite-induced decrease in heme oxygenase-1 (HO-1) protein levels and an increase in catalase (CAT) and superoxide dismutase 1 (SOD1) protein expression, suggesting a potential therapeutic avenue for JP4-039 in mitigating sulfite-induced oxidative stress in the brain. The concentrations of HO-1, CAT, and SOD1 were analyzed by Western blot.

The striatum, a key region in the brain involved in motor control and reward processing, also plays a significant role in cardiovascular regulation [88]. It receives input from various brain regions involved in autonomic control, including the hypothalamus and brainstem, and contributes to the modulation of cardiovascular function through its connections with the autonomic nervous system. Dysfunction within the striatum has been implicated in the pathogenesis of cardiovascular disease such as hypertension, heart failure, and arrhythmia [89]. Altered neurotransmitter signaling pathways and dysregulated striatal activity can disrupt normal cardiovascular regulation, leading to increased sympathetic nervous system activity, impaired baroreflex sensitivity, and elevated blood pressure [90]. Additionally, oxidative stress-induced damage in the striatum may further exacerbate cardiovascular dysfunction by promoting endothelial dysfunction, vascular inflammation, and oxidative damage in the cardiovascular system [91].

### 3.3. CK Activity Maintenance

The impact of JP4-039 on creatine kinase (CK) activity in the striatum was also examined in a study focusing on sulfite-induced alterations. In this study, a rat model was used, with JP4-039 administered intraperitoneally at 3 μg/g 24 h and 5.0 μg/g 2 h prior to sulfite injection. Here, it was observed that sulfite injection led to a decrease in CK activity [9]. However, pretreatment with a high dose of JP4-039 resulted in the preservation of CK activity, while the low-dose regimen did not show similar effects.

CK plays a pivotal role in myocardial energetics by catalyzing the reversible transfer of a phosphate group between ATP and creatine, thereby generating phosphocreatine (PCr) and ADP as a rapid energy buffer during periods of high demand [92,93]. The dysregulation of CK activity is a hallmark of cardiovascular disease, including heart failure, ischemic heart disease, and cardiomyopathy, where impaired ATP regeneration contributes to contractile dysfunction and disease progression [94,95]. Alterations in CK isoform expression, such as shifts between the cytosolic muscle–brain isoform (CK-MB) and the mitochondrial isoform (CK-MT), have been linked to maladaptive remodeling and impaired cardiac function [95,96]. Reduced CK activity has also been associated with disturbances in calcium handling and excitation–contraction coupling, further worsening myocardial dysfunction [97]. Given its central role, strategies aimed at augmenting CK activity (e.g., creatine supplementation or gene therapy) have been investigated as therapeutic interventions with promising results [98].

The preservation of CK activity by JP4-039 under oxidative stress may be explained by several potential mechanisms. First, JP4-039’s mitochondrial ROS scavenging likely prevents the oxidative modification and inactivation of CK enzymes, which are particularly vulnerable to redox imbalance [99]. Second, by stabilizing mitochondrial membrane integrity and limiting peroxidative damage, JP4-039 may protect the close spatial and functional coupling between CK-MT and adenine nucleotide translocase, thereby ensuring efficient energy transfer from mitochondria to cytosolic ATPases [100]. Third, by maintaining ATP buffering capacity, JP4-039 could indirectly preserve calcium handling and excitation–contraction coupling, which are highly dependent on an adequate energy supply [101]. Together, these mechanisms suggest that JP4-039 not only reduces oxidative stress but also sustains myocardial bioenergetics, providing a plausible explanation for its observed cardioprotective effects in conditions such as heart failure, ischemic heart disease, and cardiomyopathy.

### 3.4. MAPK Phosphorylation and Protein Content Maintenance

In a study investigating the impact of sulfite-induced alterations on MAPK phosphorylation and protein content using a rat model, JP4-039 exhibited notable effects on these signaling pathways [9]. JP4-039 was administered intraperitoneally at 3 μg/g 24 h and 5.0 μg/g 2 h prior to sulfite injection. The concentrations of phosphorylated p38 (p-p38), p38, phosphorylated extracellular signal-regulated kinase (p-ERK), extracellular signal-regulated kinase (ERK), phosphorylated c-Jun N-terminal kinase (p-JNK), c-Jun N-terminal kinase (JNK), and β-actin, among other proteins, were measured through a Western blot analysis. The findings from the study revealed that sulfite exposure led to a decrease in p38 phosphorylation, a change that was prevented by JP4-039 treatment. Conversely, sulfite injection increased ERK1/2 phosphorylation, which was attenuated by JP4-039 [9]. Intriguingly, JP4-039 treatment alone increased the level of total p38 protein.

**Figure 3:**
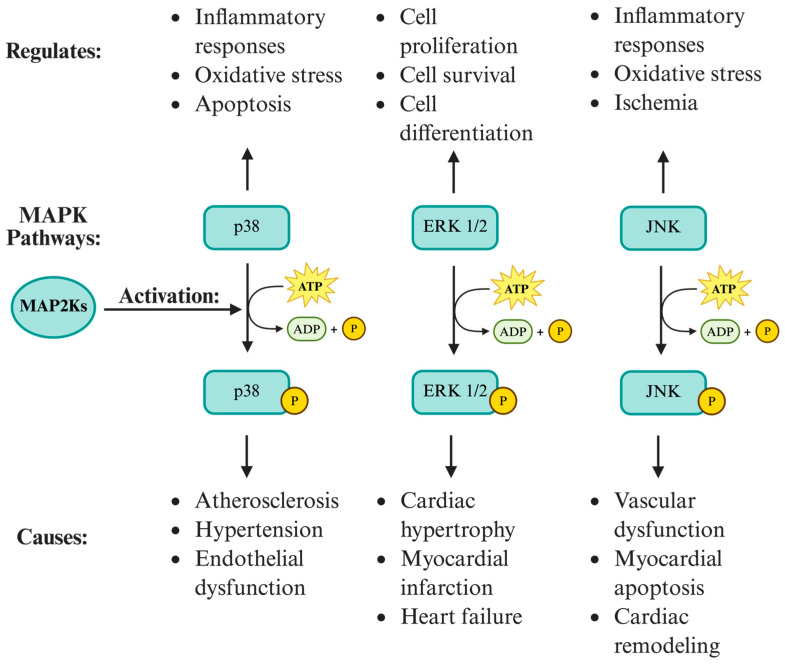
MAPK (mitogen-activated protein kinase) signaling pathways (p38, ERK1/2, and JNK) in cardiovascular disease. These cascades regulate inflammation, apoptosis, and remodeling, contributing to hypertension, atherosclerosis, and myocardial infarction. JP4-039’s ability to modulate the phosphorylation of these kinases suggests potential cardioprotective effects. Created with BioRender.com. MAP2Ks, mitogen-activated protein kinase kinases; MAPK, mitogen-activated protein kinase; p38, p38 mitogen-activated protein kinase; ERK1/2, extracellular signal-regulated kinases 1 and 2; JNK, c-Jun N-terminal Kinase; ATP, adenosine triphosphate; ADP, adenosine diphosphate; and P, phosphate group.

The MAPK signaling pathways, including p38, ERK1/2, and JNK, play critical roles in mediating cellular responses to various extracellular stimuli and are implicated in the pathogenesis of CVD (Figure 3) [102]. The dysregulation of MAPK signaling has been linked to the development and progression of conditions such as hypertension, atherosclerosis, myocardial infarction, and heart failure [103].

The p38 MAPK pathway is involved in the regulation of inflammatory responses, oxidative stress, and apoptosis, all of which contribute to the pathogenesis of CVD [104]. The activation of p38 MAPK has been observed in hypertensive models and atherosclerotic plaques, where it promotes vascular inflammation, smooth muscle cell proliferation, and endothelial dysfunction through various mechanisms [105]. Within the arterial intima, p38 MAPK activation stimulates the NF-κB pathway, facilitating the recruitment of circulating monocytes and macrophages into the vessel wall, promoting the initiation and progression of atherosclerotic lesions [106,107,108]. Moreover, p38 MAPK signaling induces the proliferation of vascular smooth muscle cells (VSMCs), resulting in intimal thickening and fibrous cap formation [109,110]. Additionally, activated p38 MAPK impairs endothelial function by reducing the bioavailability of nitric oxide (NO) by inhibiting endothelial nitric oxide synthase (eNOS) and promoting ROS generation [111]. The inhibition of p38 MAPK has been shown to attenuate these processes and improve cardiovascular outcomes in preclinical studies [112]. Specifically, the inhibition of p38 has been shown to decrease mitochondrial swelling, prevent ultrastructure alterations, mitigate mitochondrial membrane depolarization, decrease calcium influx, and reduce oxidative stress, thereby preserving mitochondrial integrity and function during IR [113,114,115]. Since JP4-039 was found to prevent the phosphorylation, and subsequent activation, of p38 MAPK, it is reasonable to expect that JP4-039 would inhibit p38 and improve cardiovascular outcomes [9].

Similarly, the ERK1/2 MAPK pathway regulates cell proliferation, survival, and differentiation, and is implicated in the pathophysiology of CVD [116]. Dysregulated ERK1/2 signaling has previously been shown to be a key factor in the development of cardiac hypertrophy, myocardial infarction, and heart failure [117]. Increased ERK1/2 activation has been observed in response to hypertrophic stimuli and ischemic injury, contributing to adverse cardiac remodeling and dysfunction [118]. The inhibition of ERK1/2 signaling has been shown to ameliorate cardiac hypertrophy and improve cardiac function in experimental models of heart disease [119]. The findings from a study showed that JP4-039 attenuated ERK1/2 phosphorylation, meaning that ERK1/2 signaling is inhibited, therefore suggesting that JP4-039 would indirectly improve cardiac function [9].

The JNK MAPK pathway is activated in response to cellular stressors such as oxidative stress, inflammation, and ischemia, and is involved in the pathogenesis of CVD [120]. Previous research has shown that JNK activation can lead to the development of vascular dysfunction, myocardial apoptosis, and cardiac remodeling in various cardiovascular pathologies [121]. The inhibition of JNK signaling has been shown to attenuate these processes and improve cardiovascular outcomes in experimental models [122]. Specifically, the inhibition of different JNK isoforms has been consistently shown to decrease myocardial infarct size and attenuate cardiomyocyte apoptosis [123]. Prior investigations into the effects of AS601245, a nonpeptide JNK inhibitor, on infarct size caused by myocardial ischemia–reperfusion (IR) in anaesthetized rats also revealed significant reductions in infarct size at 4.5 mg/kg (44%) and 15 mg/kg i.v. (40.3%) [124]. Although the study that focused on the impact of sulfite-induced alterations along with JP4-039 did not show a significant change in total JNK levels in response to sulfite injection or JP4-039 treatment, there was a trend towards an increase in phosphorylation with JP4-039 treatment, suggesting that JP4-039 could play a role in cardiomyocyte apoptosis and improve cardiovascular outcomes through its role in JNK signaling [9,123].

### 3.5. Apoptotic Regulation

Apoptosis, or programmed cell death, plays a critical role in CVD, contributing to tissue damage, vascular dysfunction, and organ failure [125]. The proteins implicated in the apoptosis cascade, such as glycogen synthase kinase-3 beta (GSK-3β), B-cell lymphoma 2 related ovarian killer (Bok), caspase-3, caspase-9, and B-cell lymphoma-extra large (Bcl-xL), are key regulators of apoptotic pathways and contribute to the pathogenesis of CVD [126].

In a study that examined the effects of sulfite-induced alterations and JP4-039 on rat striata, an intraperitoneal injection of JP4-039 has also been shown to effectively mitigate sulfite-induced apoptosis in the striatum by modulating key proteins involved in the apoptosis cascade [9]. Western blot analysis revealed that sulfite exposure significantly increased the levels of GSK-3β, Bok, and cleaved caspase-3, indicative of apoptotic cell death. However, pretreatment with JP4-039 prevented or mitigated these changes, suggesting its potential therapeutic efficacy in attenuating sulfite-induced neurotoxicity [9].

GSK-3β, a serine/threonine kinase, is involved in multiple signaling pathways regulating cell survival and apoptosis [127]. Prior studies have revealed the involvement of dysregulated GSK-3β activity in the development of atherosclerosis, myocardial infarction, and heart failure [128]. The inhibition of GSK-3β has been shown to confer cardioprotective effects by reducing apoptosis and promoting cell survival in experimental models of CVD [129]. A study on rat striata found that JP4-039 significantly reduced GSK-3β levels, counteracting the effects of sulfite injections, meaning that JP4-039 could promote cell survival in CVD [9,130].

Bok, a pro-apoptotic member of the B-cell lymphoma 2 (Bcl-2) protein family, promotes mitochondrial outer membrane permeabilization and apoptosis [131]. Increased expression of Bok has been observed in cardiac IR injury and heart failure, where it contributes to cardiomyocyte death and myocardial dysfunction [132]. Targeting Bok-mediated apoptosis may represent a therapeutic strategy for preventing myocardial injury and preserving cardiac function in CVD, and JP4-039 was found to significantly reduce Bok levels after sulfite injection in rat striate, suggesting that it might be a good therapeutic strategy [9]. Unlike Bok, Bcl-xL, an anti-apoptotic member of the Bcl-2 protein family, promotes cell survival by inhibiting mitochondrial outer membrane permeabilization and preventing cytochrome c release, suggesting that its dysregulation would result in effects like those seen with increased Bok expression: myocardial dysfunction and heart failure [133,134]. Although the results from a study on rat striata did not show a significant difference in Bcl-xL levels after both sulfite injection and JP4-039 administration, there was a trend towards a decreased level of Bcl-xL when both sulfite and JP4-039 were administered, as opposed to just a sulfite injection, suggesting that further research might reveal more mechanistic details [9].

Caspase-3, a key executioner caspase, and caspase-9, an initiator caspase, are both involved in triggering the mitochondrial apoptotic pathway, contributing to myocardial infarction and cardiac remodeling [135,136,137]. Accordingly, studies have revealed that the inhibition of these caspases can attenuate cardiomyocyte apoptosis and improve cardiac function [138]. Although a study on JP4-039- and sulfite-induced alterations did not show any significant changes in caspase-3 or caspase-9 levels after JP4-039 administration, it interestingly showed a significant decrease in the ratio of cleaved caspase-3/total caspase-3, suggesting that JP4-039 decreases the amount of caspase-3 that is cleaved and activated [9]. This suggests that JP4-039 can inhibit caspase-3 and improve cardiac function [138].

The observed effects of JP4-039 on mitigating sulfite-induced alterations in apoptosis cascades highlight its potential as a therapeutic agent for mitigating oxidative stress-induced cellular damage, not only in the striatum but also in cardiovascular tissues. By modulating key proteins involved in apoptosis, such as GSK-3β, Bok, and cleaved caspase-3, JP4-039 demonstrates its ability to intervene in pathways leading to cell death. Moreover, given the established role of apoptosis in atherosclerosis, myocardial infarction, and heart failure, targeting apoptotic regulation remains a critical therapeutic strategy.

Beyond apoptosis, recent findings also implicate JP4-039 in regulating ferroptosis, another form of programmed cell death driven by lipid peroxidation and iron overload. A recent study demonstrated that JP4-039 effectively attenuates ferroptosis by preserving mitochondrial redox homeostasis, reducing lipid peroxides, and maintaining the activity of glutathione peroxidase 4 (GPX4), a key ferroptosis regulator [139]. By targeting both apoptosis and ferroptosis pathways, JP4-039 appears to confer broader cytoprotective effects, further underscoring its potential as a treatment for cardiovascular disorders.

## 4. JP4-039 in Radiation Oncology

JP4-039 has been extensively investigated as a radioprotective agent because of its ability to mitigate radiation-induced oxidative stress, mitochondrial dysfunction, and apoptosis. Ionizing radiation generates high levels of mitochondrial ROS, leading to DNA damage, mitochondrial membrane depolarization, and the activation of cell death pathways. By scavenging mitochondrial ROS and preserving electron transport chain function, JP4-039 enhances cell survival following irradiation in multiple preclinical models. For example, studies have shown that JP4-039 reduces radiation-induced apoptosis, supports potentially lethal damage repair, and limits mitochondrial swelling and depolarization in irradiated tissues [129,140,141,142,143,144].

Although these studies were not performed in cardiovascular systems, they underscore two important aspects relevant to CVD translation: first, the consistent demonstration of JP4-039’s safety and efficacy across diverse tissues (hematopoietic, neuronal, and gastrointestinal) provides confidence in its systemic tolerability; second, the ability to protect highly oxidative stress-sensitive tissues highlights its potential to safeguard the cardiovascular system, where similar ROS-driven mechanisms underlie ischemia–reperfusion injury, endothelial dysfunction, and maladaptive remodeling. Thus, while radiation oncology remains a distinct application, the radiobiological evidence strengthens the rationale for considering JP4-039 as a cardioprotective therapy.

## 5. JP4-039 in Cardiac Protection

Several studies have explored the effects of JP4-039 on cardiac tissues and models of CVD (Table 2). One study examined JP4-039’s potential in cardiac protection, using human coronary artery endothelial cells (HCAECs) and post-acute myocardial infarction (AMI) mouse hearts [145]. The results underscored JP4-039’s efficacy in improving oxidative phosphorylation (OXPHOS) in HCAECs and inducing coronary angiogenesis in AMI mouse hearts, as evidenced by the increased expression of mitochondrial complexes I and V, enhanced respiration, ATP production, and spare respiratory capacity in vitro [145]. Specifically, in the murine AMI model, JP4-039 administration through an intramyocardial injection resulted in notable enhancements in left ventricle (LV) systolic function and capillary density, emphasizing its promising role in mitigating the consequences of ischemic myocardium and supporting the notion that a mito-ROS scavenger could play a significant role in coronary angiogenesis for cardiac health.

In another study, Friend leukemia virus B (FVB) mice treated with JP4-039 after left anterior artery ligation surgery exhibited a significant reduction in infarction size by 35% and a remarkable 57% increase in left ventricular ejection fraction (EF) compared to the vehicle control group [146]. The 35% reduction in infarction size demonstrates JP4-039’s substantial limitation of myocardial damage post-ischemic injury, while the 57% increase in EF highlights its significant enhancement in post-myocardial infarction (post-MI) cardiac function, indicating that JP4-039 not only limited infarct size but also positively influenced overall cardiac pumping efficiency [149,150]. Moreover, the study revealed a noteworthy 39% increase in capillary density within the ischemic myocardium of the JP4-039-treated mice [146]. This augmentation in capillary density points towards enhanced angiogenesis, a crucial process for re-establishing blood supply to the damaged heart tissue [151]. The promotion of angiogenesis by JP4-039 underscores its potential as a therapeutic agent for cardiac recovery post-MI, as the increased capillary network facilitates improved oxygen and nutrient delivery, fostering the restoration of the injured myocardium. These findings collectively highlight the multifaceted benefits of JP4-039 in preserving cardiac structure and function following myocardial infarction.

Another study showed that HCAECs treated with JP4-039 exhibited a significant increase in the expression of mitochondrial complexes I and V, as measured by Western blot, accompanied by enhanced basal and maximal respiration, ATP production, and spare respiratory capacity compared to control vehicle-treated cells [145]. This robust improvement in oxidative phosphorylation underscores JP4-039’s potential to positively influence cellular energy dynamics. Moreover, JP4-039 demonstrated its angiogenic potential by substantially increasing tube formation in NOX2-overexpressing mouse heart endothelial cells (MHECs) and HCAECs, as well as promoting endothelial cell sprouting in mouse atrial tissues ex vivo [145]. Complementing these findings, a separate study used a mitoSox assay and found that the treatment of HCAECs with JP4-039 significantly reduced mitochondrial ROS levels [152]. These in vitro findings emphasize JP4-039’s efficacy in fostering mitochondrial health and angiogenic processes, laying a strong foundation for its translational implications in cardiac protection.

## 6. Preclinical Safety and Potential Delivery Strategies for JP4-039

### 6.1. Preclinical Safety Studies

While clinical studies using JP4-039 have yet to be conducted, results from multiple studies in murine models suggested no adverse side effects (Table 2). In a study on JP4-039 as a radiation mitigator in mice, following total-body irradiation (TBI) doses of 9.0 and 9.15 Gy, mice treated with intraperitoneal JP4-039 (10 mg/kg) did not exhibit deleterious effects on peripheral blood counts, bone marrow cellularity, or hematopoietic recovery [147]. Notably, there were no adverse impacts on serum electrolytes, liver, or renal function tests observed in JP4-039-treated mice after irradiation. The study further indicated that survival rates in mice treated with JP4-039 were comparable to irradiated controls, emphasizing the safety profile of this radiation mitigator [147]. Marrow recovery, as measured by cellularity and hematopoietic colony-forming cells, including primitive granulocyte-erythroid-megakaryocyte-monocytes (GEMMs), reached pre-irradiation levels by day 30 in the JP4-039 treated groups.

In another study, timed pregnant C57BL/6NHsd mice at E13.5 were exposed to 3 Gy of irradiation [148]. A subgroup of these irradiated mice received intravenous injections of JP4-039 24 h later (E14.5), while another subgroup of nonirradiated mice also received JP4-039. The outcomes were measured in terms of live births, pup weight at day 5 post-birth, and the number of survivors at weaning. Nonirradiated mice had a high survival rate, while pups from irradiated mothers showed decreased survival and weight [148]. However, those from irradiated mothers treated with JP4-039 exhibited significantly improved survival, weight, and developmental outcomes compared to the irradiated control group. Microscopic examinations revealed protective effects of JP4-039, mitigating radiation-induced pathological changes in various organs. The study concludes that JP4-039, administered post-irradiation, proves to be a safe and effective radiation mitigator, enhancing fetal survival, growth, and development in mice [148]. Consistent with these findings, another study further demonstrated the mitigation of fetal radiation injury from mid-gestation total-body irradiation via the maternal administration of JP4-039 [153]. Overall, multiple studies present evidence that JP4-039 displayed no adverse side effects in animal and cell models, while highlighting the importance of further clinical research on identifying the safety profile and dosing regimen of JP4-039 in clinical applications.

Beyond these preclinical observations, a more complete pharmacological profile of JP4-039 remains to be defined. Limited pharmacokinetic studies have reported rapid clearance following intravenous administration, with a plasma half-life of approximately 10 min [154]. In addition, tissue-specific uptake studies have shown maximal accumulation in the retina within 15 min after intravitreal injection, demonstrating the compound’s ability to reach and persist in metabolically active tissues sensitive to oxidative stress [58]. While these findings provide initial insights into distribution and clearance, comparable studies are lacking in cardiovascular models. Defining JP4-039’s absorption, tissue bioavailability, and metabolic fate in the heart and vasculature will be critical for optimizing dosing strategies, evaluating long-term safety, and advancing translational research in chronic cardiovascular applications.

### 6.2. Extracellular Vesicles as a Potential Delivery Mechanism for JP4-039

While JP4-039 has demonstrated promising safety and efficacy in preclinical models, optimizing its delivery method, by focusing on different transfer mechanisms, could further enhance its therapeutic potential. In recent years, researchers have increasingly focused on extracellular vesicles (EVs) due to their ability to engage in cell-to-cell communication and influence angiogenesis, offering an alternative mechanism for intercellular signaling beyond traditional drugs [155]. For example, hypoxia-conditioned extracellular vesicles (HEVs) were used as a therapy for oxidative stress in a swine model of chronic myocardial ischemia [156]. This application suggests considerable potential for further therapeutic formulation development.

## 7. Limitations and Future Directions

Although JP4-039 demonstrates promising preclinical efficacy as a mitochondria-targeted antioxidant, several limitations of the current research must be acknowledged. First, there are no human studies evaluating its safety, pharmacokinetics, or efficacy in cardiovascular disease. The available evidence is derived almost entirely from short-term animal models, which limits understanding of its effects on chronic comorbidities such as hypertension, diabetes, and metabolic syndrome. Second, variability in formulations, dosing regimens, and administration routes across preclinical studies complicates interpretation and hinders direct translation. Third, while limited pharmacokinetic studies have reported rapid clearance after intravenous administration (half-life of ~10 min) [154] and tissue-specific uptake in the retina after intravitreal injection [58], comparable data in the heart and vasculature remain unavailable. A more comprehensive understanding of absorption, tissue bioavailability, metabolism, and clearance in cardiovascular systems is essential for optimizing therapeutic application.

Future research should therefore focus on bridging these gaps through well-designed preclinical and early-phase clinical studies. Establishing safety, bioavailability, and optimal dosing in humans will be critical first steps. In parallel, novel delivery strategies, such as extracellular vesicle-based carriers, nanoparticle formulations, or targeted conjugates, may enhance tissue specificity and therapeutic efficacy. Given the multifactorial nature of cardiovascular disease, combination strategies that integrate JP4-039 with established therapies (e.g., SGLT2 inhibitors) warrant exploration of their potential synergistic benefits. Finally, expanding investigation into chronic and comorbid models will be essential to determine whether JP4-039 can move beyond acute protection to provide durable cardiovascular benefits. By addressing these limitations and future directions, JP4-039 can be advanced from a promising preclinical candidate toward clinical translation as a novel cardiovascular therapeutic.

## 8. Conclusions

Oxidative stress plays a key role in CVD, exacerbating conditions like myocardial infarction and heart failure through the excessive generation of ROS. Amidst this pathophysiological environment, JP4-039, with its unique structural attributes, emerges as a promising candidate. Research endeavors have delved into its efficacy in mitigating oxidative stress-related damage within the cardiovascular system. Additionally, the exploration of JP4-039 extends beyond cardiovascular disease, venturing into the realm of radiation oncology and kidney disorders, where its potential as a therapeutic agent is currently being investigated [157]. Prior research suggests that understanding JP4-039’s mechanisms of action within a cardiovascular context holds promise for innovative therapeutic strategies aimed at combating the oxidative stress linked with different pathophysiological indicators of CVD: atherosclerosis, coronary artery disease, and arterial hypertension.

## Figures and Tables

**Table 1:** Mitochondrial-target antioxidants.

Antioxidant	Mitochondrial Specificity (Compared to Cytosol)	Molecular Weight (Da)	Animal Testing
JP4-039	20-fold to 30-fold [56]	424.60 [57]	- Reduced apoptosis and inflammatory cell migration in irradiated mouse retina [58] - Increased coronary vascular density in ischemic myocardium, improved ATP synthesis, and improved cardiac function post-myocardial infarction in mice [59]
XJB-5-131	600-fold [60]	959.2 [61]	- Reduced motor decline and weight loss, and enhanced neuronal survival and mitochondrial function in mice with Huntington’s disease [62] - Attenuated respiratory dysfunction and improved cardiac function recovery post-ischemia–reperfusion (IR) injury [63]
MitoQ	100-fold to 1000-fold [64]	678.8 [61]	- Decreased heart dysfunction, cell death, and mitochondrial damage after ischemia–reperfusion in rats [65] - Improved endothelial function and reduced cardiac hypertrophy in stroke-prone spontaneously hypertensive rats [66]
MitoTEMPO	1000-fold [67]	511 [61]	- Reduced renal oxidative stress and dysfunction in a rat model of fecal peritonitis [68] - Increased survival and reduced tumor incidence and multiplicity in mice with hepatocarcinogenesis [69]
SkQ1	1000-fold [70]	617.6 [61]	- Increased dopamine quantity and decreased signs of sensory–motor deficiency in mice with Parkinson’s [71] - Lowered blood glucose levels and increased expression of microRNA markers determining the function of β-cells in rats induced with diabetes [72]
SS-31	5000-fold [73]	639.802 [74]	- Improved mitochondrial respiratory capacity and ameliorated mitochondrial dysfunction in mice with TAFAZZIN knockdown to simulate Barth Syndrome [75] - Improved cardiac function and reduced myocardial interstitial fibrosis in a mouse model of pressure-overloaded heart failure [76]

**Table 2:** Summary of studies exploring JP4-039’s effects on cardiac tissues and CVD models and its preclinical safety.

Study	Animal Model	Sample Size	Major Findings
Mitochondrial ROS scavenger JP4-039 improves mitochondrial oxidative phosphorylation and induces angiogenesis in murine and human coronary artery and atrial endothelial cells	Human coronary artery endothelial cells (HCAECs) and post-acute myocardial infarction (AMI) mouse hearts	16 (8 for JP4-039 and 8 for vehicle injection)	JP4-039 improves oxidative phosphorylation in HCAECs and induces coronary angiogenesis in mouse hearts. It also increases the expression of mitochondrial complexes I and V, and increases basal and maximal respiration, adenosine triphosphate (ATP) production, and spare respiratory capacity [145]
Mitochondrial-targeted ROS scavenger JP4-039 induces coronary angiogenesis in the improvement of cardiac function in post-myocardial infarction (MI) animals	Friend leukemia virus B (FVB) mice	16 (8 for JP4-039 and 8 for vehicle injection)	JP4-039 significantly reduces infarction size and increases left ventricular ejection fraction (EF) [146]
Radiobiologic effects of GS-nitroxide (JP4-039) on the hematopoietic syndrome	Female C57BL/6HNsd irradiated mice	Most groups included 7 animals, with some control or low-dose groups including 5–9 animals	JP4-039 did not exhibit deleterious effects on peripheral blood counts, bone marrow cellularity, or hematopoietic recovery [147]
Small-molecule GS-nitroxide radiation mitigates JP4-039/F14 is safe and effective in pregnant E13.5 mice	Pregnant C57BL/6NHsd irradiated mice	15 (distributed across 5 groups)	JP4-039 significantly improved survival, weight, and developmental outcomes [148]

## Data Availability

No new data were created or analyzed in this study. Data sharing is not applicable to this article.
